# 2-Chloro­ethyl 2-(2-chloro­phen­yl)-2-(4,5,6,7-tetrahydro­thieno[3,2-*c*]pyridin-5-yl)acetate

**DOI:** 10.1107/S1600536810046908

**Published:** 2010-11-17

**Authors:** Ji-Fang Chen, Ying Liu, Jing-Yang Wang, Deng-Ke Liu

**Affiliations:** a Materials Science and Engineering, Tianjin Polytechnic University, Tianjin, 300160, People’s Republic of China; b Tianjin Institute of Pharmaceutical Research, Tianjin, 300193, People’s Republic of China

## Abstract

The mol­ecular packing of the title compound, C_17_H_17_Cl_2_NO_2_S, is stabilized by weak C—H⋯O and C—H⋯Cl inter­actions. The ester chain is almost planar with a mean deviation of 0.0605 Å and makes dihedral angles of 71.60 (4) and 74.70 (8)° with the benzene ring and the thio­phene ring, respectively. The benzene and thio­phene rings make a dihedral angle of 84.22 (7)°.

## Related literature

The title compound is a derivative of clopidogrel. For background to the bioactivity and applications of the anti­platelet agent clopidogrel, see, for example, Gurbel & Tantry (2007 ▶); Muller *et al.* (2003 ▶); Savi *et al.* (1994 ▶); Sharis *et al.* (1998 ▶). For the synthesis of other derivatives with thienopyridine, see: Aubert *et al.* (1985 ▶); Bipin *et al.* (2002 ▶); Bouisset & Radisson (1991 ▶).
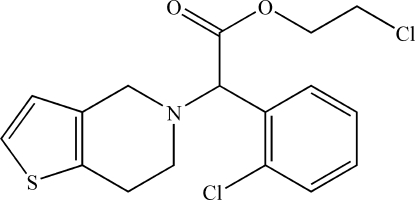


## Experimental

### 

#### Crystal data

C_17_H_17_Cl_2_NO_2_S
*M* *_r_* = 370.28
Monoclinic, 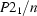

*a* = 9.689 (1) Å
*b* = 11.2670 (12) Å
*c* = 15.5670 (16) Å
β = 100.509 (8)°
*V* = 1670.9 (3) Å^3^
*Z* = 4
Cu *K*α radiation
μ = 4.73 mm^−1^
*T* = 113 K
0.26 × 0.24 × 0.20 mm

#### Data collection

Rigaku Saturn diffractometer
Absorption correction: multi-scan (*CrystalClear*; Rigaku/MSC, 2005 ▶) *T*_min_ = 0.373, *T*_max_ = 0.451
18109 measured reflections
3203 independent reflections
2974 reflections with *I* > 2σ(*I*)
*R*_int_ = 0.065

#### Refinement

*R*[*F* ^2^ > 2σ(*F* ^2^)] = 0.037
*wR*(*F* ^2^) = 0.107
*S* = 1.09
3203 reflections
210 parameters
H-atom parameters constrained
Δρ_max_ = 0.55 e Å^−3^
Δρ_min_ = −0.50 e Å^−3^

### 

Data collection: *CrystalClear* (Rigaku/MSC, 2005 ▶); cell refinement: *CrystalClear*; data reduction: *CrystalClear*; program(s) used to solve structure: *SHELXS97* (Sheldrick, 2008 ▶); program(s) used to refine structure: *SHELXL97* (Sheldrick, 2008 ▶); molecular graphics: *SHELXTL* (Sheldrick, 2008 ▶); software used to prepare material for publication: *CrystalStructure* (Rigaku/MSC, 2005 ▶).

## Supplementary Material

Crystal structure: contains datablocks global, I. DOI: 10.1107/S1600536810046908/fk2029sup1.cif

Structure factors: contains datablocks I. DOI: 10.1107/S1600536810046908/fk2029Isup2.hkl

Additional supplementary materials:  crystallographic information; 3D view; checkCIF report

## Figures and Tables

**Table 1:** Hydrogen-bond geometry (Å, °)

*D*—H⋯*A*	*D*—H	H⋯*A*	*D*⋯*A*	*D*—H⋯*A*
C7—H7a⋯O1	0.99	2.53	3.140 (2)	120
C8—H8⋯Cl1	1.00	2.59	3.042 (2)	107

**Fig. 1.**
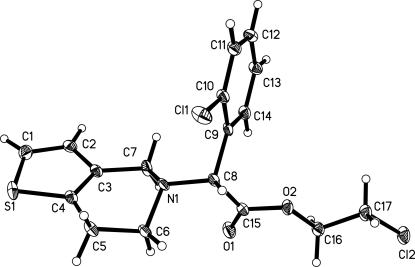
The molecular structure of (I), displacement ellipsoids are drawn at the 50% probability level.

## supplementary crystallographic information

### Comment

Clopidogrel, a thienopyridine class inhibitor of P2Y12 ADP platelet receptor, has been found to be particularly useful in the treatment of coronary artery disease, peripheral vascular disease, and cerebrovascular disease (Aubert *et al.*, 1985; Bipin *et al.*, 2002; Bouisset & Radisson, 1991; Muller *et al.*, 2003; Savi, *et al.*,1994; Gurbel & Tantry, 2007; Sharis *et al.*, 1998). The crystal structure of the title compound, 2-Chloroethyl 2-(2-chlorophenyl)-2-(6,7-dihydro thieno[3,2-*c*]pyridin-5(*4H*)-yl)acetate (I), a derivative of clopidogrel, is reported here.

As shown in Fig. 1, the benzene ring, the ester chain and the thienopyridine group are all linked to C8 and a molecular chiral center is formed. The ester chain(C15/C16/C17/O1/O2/Cl2) is almost planar, the mean deviation from the plane is 0.0605 Å. The dihedral angles formed between the benzene ring plane (A), the ester chain plane (B) and the thiophene ring plane (C) are 71.60 (4) ° (A/B), 74.70 (8) ° (B/C) and 84.22 (7) ° (A/C), respectively. The packing is consolidated by C—H···O and C—H···Cl interactions, see Table 1.

### Experimental

(I) was prepared from α-bromo(2-chloro)phenyl acetic acid and 4,5,6,7-tetrahydro thieno[3,2-*c*] pyridin by esterification and substitution reaction. Colourless crystals (m.p. 91.8–92.8°C) were obtained in a yield of 93.7%. Single crystals were grown from hexane-ethyl acetate (1:1) solution.

### Refinement

All the H atoms were positioned geometrically and refined as riding atoms, with C8—H8=1.00 Å, C—H=0.95Å (for the other CH groups), and 0.99Å (CH2), *U*_iso_ = 1.2*U*_eq_(C).

### Crystal data

**Table:**

C_17_H_17_Cl_2_NO_2_S	*F*(000) = 768
*M**_r_* = 370.28	*D*_x_ = 1.472 Mg m^−^^3^
Monoclinic, *P*2_1_/*n*	Cu *K*α radiation, λ = 1.54187 Å
Hall symbol: -P 2yn	Cell parameters from 2162 reflections
*a* = 9.689 (1) Å	θ = 27.6–72.0°
*b* = 11.2670 (12) Å	µ = 4.73 mm^−^^1^
*c* = 15.5670 (16) Å	*T* = 113 K
β = 100.509 (8)°	Prism, colorless
*V* = 1670.9 (3) Å^3^	0.26 × 0.24 × 0.20 mm
*Z* = 4	

### Data collection

**Table:**

Rigaku Saturn diffractometer	3203 independent reflections
Radiation source: fine-focus sealed tube	2974 reflections with *I* > 2σ(*I*)
multilayer	*R*_int_ = 0.065
Detector resolution: 14.63 pixels mm^-1^	θ_max_ = 72.6°, θ_min_ = 4.9°
ω scans	*h* = −11→11
Absorption correction: multi-scan (*CrystalClear*; Rigaku/MSC, 2005)	*k* = −13→13
*T*_min_ = 0.373, *T*_max_ = 0.451	*l* = −19→15
18109 measured reflections	

### Refinement

**Table:**

Refinement on *F*^2^	Secondary atom site location: difference Fourier map
Least-squares matrix: full	Hydrogen site location: inferred from neighbouring sites
*R*[*F*^2^ > 2σ(*F*^2^)] = 0.037	H-atom parameters constrained
*wR*(*F*^2^) = 0.107	*w* = 1/[σ^2^(*F*_o_^2^) + (0.0633*P*)^2^ + 0.6161*P*] where *P* = (*F*_o_^2^ + 2*F*_c_^2^)/3
*S* = 1.09	(Δ/σ)_max_ = 0.001
3203 reflections	Δρ_max_ = 0.55 e Å^−^^3^
210 parameters	Δρ_min_ = −0.50 e Å^−^^3^
0 restraints	Extinction correction: *SHELXL*, Fc^*^=kFc[1+0.001xFc^2^λ^3^/sin(2θ)]^-1/4^
Primary atom site location: structure-invariant direct methods	Extinction coefficient: 0.0023 (4)

### Special details

**Table:**

Geometry. All e.s.d.'s (except the e.s.d. in the dihedral angle between two l.s. planes) are estimated using the full covariance matrix. The cell e.s.d.'s are taken into account individually in the estimation of e.s.d.'s in distances, angles and torsion angles; correlations between e.s.d.'s in cell parameters are only used when they are defined by crystal symmetry. An approximate (isotropic) treatment of cell e.s.d.'s is used for estimating e.s.d.'s involving l.s. planes.
Refinement. Refinement of *F*^2^ against ALL reflections. The weighted *R*-factor *wR* and goodness of fit *S* are based on *F*^2^, conventional *R*-factors *R* are based on *F*, with *F* set to zero for negative *F*^2^. The threshold expression of *F*^2^ > σ(*F*^2^) is used only for calculating *R*-factors(gt) *etc*. and is not relevant to the choice of reflections for refinement. *R*-factors based on *F*^2^ are statistically about twice as large as those based on *F*, and *R*- factors based on ALL data will be even larger.

### Fractional atomic coordinates and isotropic or equivalent isotropic displacement parameters (Å^2^)

**Table:**

	*x*	*y*	*z*	*U*_iso_*/*U*_eq_	
Cl1	0.04822 (5)	0.57741 (4)	0.11386 (3)	0.02277 (16)	
Cl2	0.76621 (5)	0.81053 (4)	0.43330 (3)	0.02378 (16)	
S1	−0.27359 (5)	0.25362 (4)	0.37037 (3)	0.02083 (16)	
O1	0.32239 (15)	0.54792 (13)	0.45403 (9)	0.0225 (3)	
O2	0.39640 (14)	0.66198 (12)	0.35279 (9)	0.0174 (3)	
N1	0.08010 (16)	0.47629 (13)	0.32205 (10)	0.0130 (3)	
C1	−0.1656 (2)	0.13256 (17)	0.36824 (13)	0.0210 (4)	
H1	−0.1959	0.0524	0.3685	0.025*	
C2	−0.0317 (2)	0.16624 (17)	0.36612 (13)	0.0179 (4)	
H2	0.0432	0.1120	0.3656	0.022*	
C3	−0.01587 (19)	0.29199 (16)	0.36478 (12)	0.0144 (4)	
C4	−0.13725 (19)	0.35097 (16)	0.36683 (12)	0.0153 (4)	
C5	−0.1531 (2)	0.48332 (16)	0.36218 (13)	0.0176 (4)	
H5A	−0.2034	0.5113	0.4083	0.021*	
H5B	−0.2085	0.5066	0.3048	0.021*	
C6	−0.0070 (2)	0.53980 (16)	0.37471 (13)	0.0169 (4)	
H6A	−0.0154	0.6242	0.3568	0.020*	
H6B	0.0376	0.5364	0.4372	0.020*	
C7	0.11670 (19)	0.35649 (16)	0.35549 (14)	0.0175 (4)	
H7A	0.1803	0.3613	0.4129	0.021*	
H7B	0.1657	0.3130	0.3146	0.021*	
C8	0.19666 (18)	0.54645 (15)	0.30224 (12)	0.0139 (4)	
H8	0.1549	0.6221	0.2756	0.017*	
C9	0.26203 (18)	0.48536 (15)	0.23238 (12)	0.0132 (4)	
C10	0.19866 (18)	0.49132 (16)	0.14532 (12)	0.0143 (4)	
C11	0.2530 (2)	0.43267 (17)	0.08043 (13)	0.0175 (4)	
H11	0.2070	0.4375	0.0212	0.021*	
C12	0.3751 (2)	0.36703 (16)	0.10307 (13)	0.0179 (4)	
H12	0.4127	0.3261	0.0592	0.021*	
C13	0.44201 (19)	0.36086 (16)	0.18876 (13)	0.0164 (4)	
H13	0.5268	0.3171	0.2039	0.020*	
C14	0.38540 (19)	0.41882 (15)	0.25317 (13)	0.0155 (4)	
H14	0.4314	0.4131	0.3124	0.019*	
C15	0.31009 (19)	0.58238 (15)	0.37984 (13)	0.0160 (4)	
C16	0.5174 (2)	0.69884 (17)	0.41623 (13)	0.0188 (4)	
H16A	0.5675	0.6293	0.4458	0.023*	
H16B	0.4892	0.7517	0.4608	0.023*	
C17	0.6085 (2)	0.76441 (17)	0.36247 (14)	0.0202 (4)	
H17A	0.5577	0.8345	0.3342	0.024*	
H17B	0.6316	0.7118	0.3162	0.024*	

### Atomic displacement parameters (Å^2^)

**Table:**

	*U*^11^	*U*^22^	*U*^33^	*U*^12^	*U*^13^	*U*^23^
Cl1	0.0140 (2)	0.0368 (3)	0.0168 (3)	0.00997 (17)	0.00123 (19)	0.00264 (18)
Cl2	0.0144 (3)	0.0303 (3)	0.0259 (3)	−0.00757 (16)	0.0018 (2)	0.00000 (18)
S1	0.0121 (3)	0.0269 (3)	0.0237 (3)	−0.00480 (16)	0.0040 (2)	0.00486 (18)
O1	0.0192 (7)	0.0317 (7)	0.0173 (8)	−0.0060 (6)	0.0050 (6)	0.0020 (6)
O2	0.0138 (6)	0.0248 (7)	0.0131 (7)	−0.0073 (5)	0.0014 (5)	0.0002 (5)
N1	0.0113 (7)	0.0173 (7)	0.0119 (8)	−0.0005 (6)	0.0062 (6)	0.0000 (6)
C1	0.0235 (10)	0.0211 (9)	0.0194 (11)	−0.0054 (7)	0.0063 (8)	0.0003 (7)
C2	0.0217 (10)	0.0208 (9)	0.0126 (10)	−0.0013 (7)	0.0065 (8)	0.0001 (7)
C3	0.0136 (9)	0.0210 (9)	0.0090 (9)	−0.0004 (7)	0.0033 (7)	0.0018 (7)
C4	0.0141 (9)	0.0241 (9)	0.0079 (9)	−0.0021 (7)	0.0027 (7)	0.0012 (7)
C5	0.0148 (9)	0.0225 (9)	0.0174 (10)	0.0024 (7)	0.0077 (8)	0.0037 (7)
C6	0.0154 (9)	0.0186 (8)	0.0191 (10)	0.0005 (7)	0.0100 (8)	−0.0004 (7)
C7	0.0101 (8)	0.0171 (8)	0.0262 (11)	0.0005 (6)	0.0058 (8)	0.0031 (7)
C8	0.0111 (8)	0.0172 (8)	0.0143 (9)	−0.0011 (6)	0.0049 (7)	−0.0002 (7)
C9	0.0093 (8)	0.0167 (8)	0.0143 (9)	−0.0025 (6)	0.0044 (7)	0.0013 (6)
C10	0.0103 (8)	0.0205 (8)	0.0123 (10)	−0.0005 (6)	0.0026 (7)	0.0007 (7)
C11	0.0135 (9)	0.0243 (9)	0.0145 (10)	−0.0015 (7)	0.0020 (7)	−0.0003 (7)
C12	0.0169 (9)	0.0207 (9)	0.0174 (10)	0.0008 (7)	0.0068 (8)	−0.0030 (7)
C13	0.0131 (9)	0.0204 (9)	0.0161 (10)	0.0024 (7)	0.0036 (7)	0.0001 (7)
C14	0.0114 (9)	0.0190 (9)	0.0159 (10)	−0.0006 (6)	0.0023 (7)	0.0013 (7)
C15	0.0124 (9)	0.0183 (9)	0.0188 (11)	−0.0002 (6)	0.0068 (8)	−0.0017 (7)
C16	0.0137 (9)	0.0262 (10)	0.0162 (10)	−0.0057 (7)	0.0024 (8)	−0.0035 (7)
C17	0.0140 (9)	0.0240 (9)	0.0222 (11)	−0.0049 (7)	0.0022 (8)	−0.0004 (8)

### Geometric parameters (Å, °)

**Table:**

Cl1—C10	1.7450 (18)	C6—H6B	0.9900
Cl2—C17	1.791 (2)	C7—H7A	0.9900
S1—C1	1.723 (2)	C7—H7B	0.9900
S1—C4	1.7256 (18)	C8—C9	1.520 (2)
O1—C15	1.204 (2)	C8—C15	1.532 (3)
O2—C15	1.345 (2)	C8—H8	1.0000
O2—C16	1.449 (2)	C9—C10	1.384 (3)
N1—C8	1.457 (2)	C9—C14	1.398 (3)
N1—C6	1.466 (2)	C10—C11	1.389 (3)
N1—C7	1.467 (2)	C11—C12	1.385 (3)
C1—C2	1.358 (3)	C11—H11	0.9500
C1—H1	0.9500	C12—C13	1.374 (3)
C2—C3	1.426 (3)	C12—H12	0.9500
C2—H2	0.9500	C13—C14	1.390 (3)
C3—C4	1.356 (3)	C13—H13	0.9500
C3—C7	1.506 (2)	C14—H14	0.9500
C4—C5	1.499 (3)	C16—C17	1.515 (3)
C5—C6	1.532 (3)	C16—H16A	0.9900
C5—H5A	0.9900	C16—H16B	0.9900
C5—H5B	0.9900	C17—H17A	0.9900
C6—H6A	0.9900	C17—H17B	0.9900
			
C1—S1—C4	91.80 (9)	C9—C8—C15	110.57 (14)
C15—O2—C16	116.73 (15)	N1—C8—H8	106.2
C8—N1—C6	113.66 (14)	C9—C8—H8	106.2
C8—N1—C7	115.33 (14)	C15—C8—H8	106.2
C6—N1—C7	112.16 (14)	C10—C9—C14	117.43 (17)
C2—C1—S1	111.44 (15)	C10—C9—C8	120.70 (16)
C2—C1—H1	124.3	C14—C9—C8	121.85 (17)
S1—C1—H1	124.3	C9—C10—C11	122.00 (17)
C1—C2—C3	112.55 (17)	C9—C10—Cl1	120.04 (14)
C1—C2—H2	123.7	C11—C10—Cl1	117.94 (15)
C3—C2—H2	123.7	C12—C11—C10	119.25 (19)
C4—C3—C2	113.01 (17)	C12—C11—H11	120.4
C4—C3—C7	121.62 (16)	C10—C11—H11	120.4
C2—C3—C7	125.23 (16)	C13—C12—C11	120.25 (18)
C3—C4—C5	124.61 (16)	C13—C12—H12	119.9
C3—C4—S1	111.19 (14)	C11—C12—H12	119.9
C5—C4—S1	124.14 (14)	C12—C13—C14	119.86 (17)
C4—C5—C6	108.82 (15)	C12—C13—H13	120.1
C4—C5—H5A	109.9	C14—C13—H13	120.1
C6—C5—H5A	109.9	C13—C14—C9	121.20 (18)
C4—C5—H5B	109.9	C13—C14—H14	119.4
C6—C5—H5B	109.9	C9—C14—H14	119.4
H5A—C5—H5B	108.3	O1—C15—O2	123.85 (18)
N1—C6—C5	109.83 (15)	O1—C15—C8	127.06 (17)
N1—C6—H6A	109.7	O2—C15—C8	109.08 (15)
C5—C6—H6A	109.7	O2—C16—C17	104.11 (16)
N1—C6—H6B	109.7	O2—C16—H16A	110.9
C5—C6—H6B	109.7	C17—C16—H16A	110.9
H6A—C6—H6B	108.2	O2—C16—H16B	110.9
N1—C7—C3	108.85 (15)	C17—C16—H16B	110.9
N1—C7—H7A	109.9	H16A—C16—H16B	109.0
C3—C7—H7A	109.9	C16—C17—Cl2	108.60 (15)
N1—C7—H7B	109.9	C16—C17—H17A	110.0
C3—C7—H7B	109.9	Cl2—C17—H17A	110.0
H7A—C7—H7B	108.3	C16—C17—H17B	110.0
N1—C8—C9	110.25 (14)	Cl2—C17—H17B	110.0
N1—C8—C15	116.67 (15)	H17A—C17—H17B	108.4
			
C4—S1—C1—C2	0.79 (17)	N1—C8—C9—C10	78.1 (2)
S1—C1—C2—C3	−0.9 (2)	C15—C8—C9—C10	−151.36 (16)
C1—C2—C3—C4	0.6 (2)	N1—C8—C9—C14	−100.15 (19)
C1—C2—C3—C7	−175.08 (19)	C15—C8—C9—C14	30.3 (2)
C2—C3—C4—C5	−177.23 (17)	C14—C9—C10—C11	0.9 (3)
C7—C3—C4—C5	−1.3 (3)	C8—C9—C10—C11	−177.49 (16)
C2—C3—C4—S1	0.0 (2)	C14—C9—C10—Cl1	−177.49 (13)
C7—C3—C4—S1	175.86 (15)	C8—C9—C10—Cl1	4.1 (2)
C1—S1—C4—C3	−0.42 (16)	C9—C10—C11—C12	−0.6 (3)
C1—S1—C4—C5	176.78 (17)	Cl1—C10—C11—C12	177.76 (14)
C3—C4—C5—C6	−12.0 (3)	C10—C11—C12—C13	−0.5 (3)
S1—C4—C5—C6	171.12 (14)	C11—C12—C13—C14	1.3 (3)
C8—N1—C6—C5	157.57 (15)	C12—C13—C14—C9	−1.0 (3)
C7—N1—C6—C5	−69.4 (2)	C10—C9—C14—C13	0.0 (3)
C4—C5—C6—N1	44.8 (2)	C8—C9—C14—C13	178.30 (16)
C8—N1—C7—C3	−174.85 (15)	C16—O2—C15—O1	5.7 (3)
C6—N1—C7—C3	52.9 (2)	C16—O2—C15—C8	−175.09 (14)
C4—C3—C7—N1	−18.0 (3)	N1—C8—C15—O1	9.3 (3)
C2—C3—C7—N1	157.41 (17)	C9—C8—C15—O1	−117.7 (2)
C6—N1—C8—C9	−167.86 (15)	N1—C8—C15—O2	−169.92 (14)
C7—N1—C8—C9	60.6 (2)	C9—C8—C15—O2	63.05 (18)
C6—N1—C8—C15	65.0 (2)	C15—O2—C16—C17	167.79 (15)
C7—N1—C8—C15	−66.6 (2)	O2—C16—C17—Cl2	−177.94 (12)

### Hydrogen-bond geometry (Å, °)

**Table:**

*D*—H···*A*	*D*—H	H···*A*	*D*···*A*	*D*—H···*A*
C7—H7a···O1	0.99	2.53	3.140 (2)	120.0
C8—H8···Cl1	1.00	2.59	3.042 (2)	107
